# Use of ReCross to Increase Success Rate With Antegrade CTO PCI—A Multicenter Observational Study

**DOI:** 10.1002/ccd.31585

**Published:** 2025-05-19

**Authors:** Mohamed Ayoub, Abdul Mozid, Legate Philip, Xavier Millan, Jaikirshan Khatri, Claudiu Ungureanu, Johan Bennett, Chadi Ghafari, Alexandre Avran

**Affiliations:** ^1^ Clinic for General and Interventional Cardiology/Angiology Herz‐ und Diabeteszentrum NRW, Ruhr‐Universität Bochum Bad Oeynhausen Germany; ^2^ Cardio‐Respiratory Clinical Services Unit Leeds General Infirmary, The Leeds Teaching Hospitals NHS Trust Leeds UK; ^3^ Cardiology Department Hospital de la Santa Creu i Sant Pau, Biomedical Research Institute (IIB Sant Pau) Barcelona Spain; ^4^ Interventional Cardiology, Heart, Vascular, and Thoracic Institute Cleveland Clinic Foundation Cleveland Ohio USA; ^5^ Department of Cardiology CHU Helora, Jolimont La Louviére Belgium; ^6^ Department of Cardiovascular Medicine University Hospital Leuven Leuven Belgium; ^7^ Department of Cardiology University of Mons (UMONS) Mons Belgium; ^8^ Department of Cardiology CHU Helora, Site Kennedy Mons Belgium; ^9^ Valenciennes Hospital Valenciennes France

**Keywords:** antegrade dissection re‐entry, antegrade wiring, retrograde dissection re‐entry

## Abstract

**Background:**

Antegrade approach remains one of the predominant techniques for CTO PCI. Success rates have increased significantly recently due to advancements in devices and strategies, along with the hybrid algorithm. ReCross is a uniquely modified microcatheter with two over‐the‐wire lumens and three exit ports, offering significant potential for application across all facets of antegrade CTO‐PCI applications. However, there is limited published data investigating its efficacy.

**Objective:**

The objective of our study was to analyze the efficiency and outcome of the ReCross device, following an agreed step‐by‐step protocol in contemporary CTO PCI, based on our firsthand experience.

**Methods:**

During the period between June 2023 and April 2024, we evaluated patients undergoing CTO PCI at eight participating centers for scheduled clinically necessary CTO PCI with a planned primary antegrade approach using an upfront agreed ReCross strategy.

**Results:**

A total of 118 patients who underwent CTO PCI were included in the study. The mean JCTO score was 2.16 ± 0.95. The majority of cases (94%) were successfully resolved using an upfront ReCross strategy with a technical success of 96%, demonstrating a high level of effectiveness. The average procedure time (90 min) observed in our study was notably lower in comparison to other similar studies. There were no procedure‐related mortalities until hospital discharge, although one distal wire perforation complication was reported.

**Conclusion:**

Our initial experience with the ReCross microcatheter suggests in select cases with the application of a dedicated algorithm, the antegrade approach can be highly successful.

**Abbreviations**
ADR: antegrade dissection re‐entry
AWE: antegrade wire escalation
CTO: chronic total occlusion
RDR: retrograde dissection re‐entry

## 1 Introduction

The percutaneous treatment of chronic total occlusion (CTO) of coronary arteries can present significant challenges, however the past decade has witnessed a remarkable progress in percutaneous coronary intervention (PCI) techniques, with success rates now exceeding 90% in experienced centers [1, 2, 3]. These advancements are the result of both new cutting‐edge devices and refined strategies for crossing CTOs.

The introduction of the “hybrid algorithm” has significantly enhanced the success rates, safety, and efficiency of these procedures on a global scale [1, 4, 5]. Data consistently indicate that antegrade wiring (AW) serves as the predominant strategy for navigating through CTOs, particularly those of lower complexity [4]. It is imperative to acknowledge that achieving a high success rate in CTO PCI necessitates proficiency in dissection and re‐entry techniques (DART) as well as the use of retrograde approaches to achieve a comprehensive and efficacious treatment approach.

The primary objective of the AW technique is for the guidewire to puncture the proximal cap of the CTO, traverse the entire length of the CTO, and re‐enter the true lumen at the distal cap. While the intention is to remain intra‐plaque or in “true lumen” predominantly, it is not uncommon for the wire to pass in and out of the vessel extra‐plaque/sub‐intimal space during AW, often not appreciable on fluoroscopy alone [5, 6]. In cases where a wire is in the extra‐plaque space, the parallel wire technique can be used to find the true lumen. This technique involves leaving the initial wire in place to act as a visual marker, while a second wire, with a microcatheter, is advanced alongside or in contact with the original wire. Visual and tactile cues are used to guide the passage of the second wire, chosen specifically to address the failure mode of the first wire, until distal true lumen wire placement is achieved. This method, including a variation known as seesaw wiring, is a cornerstone of contemporary antegrade CTO PCI. The antegrade dissection and re‐entry (ADR) method, on the other hand, entails intentionally directing the guidewire and/or equipment into a dissection plane before re‐entering the distal vessel lumen at or beyond the distal cap. This approach is especially beneficial when dealing with lengthy or complex CTOs and can help resolve inadvertent extra plaque wire passage during AW that cannot be resolved [1, 7, 8, 9].

**Figure 1:**
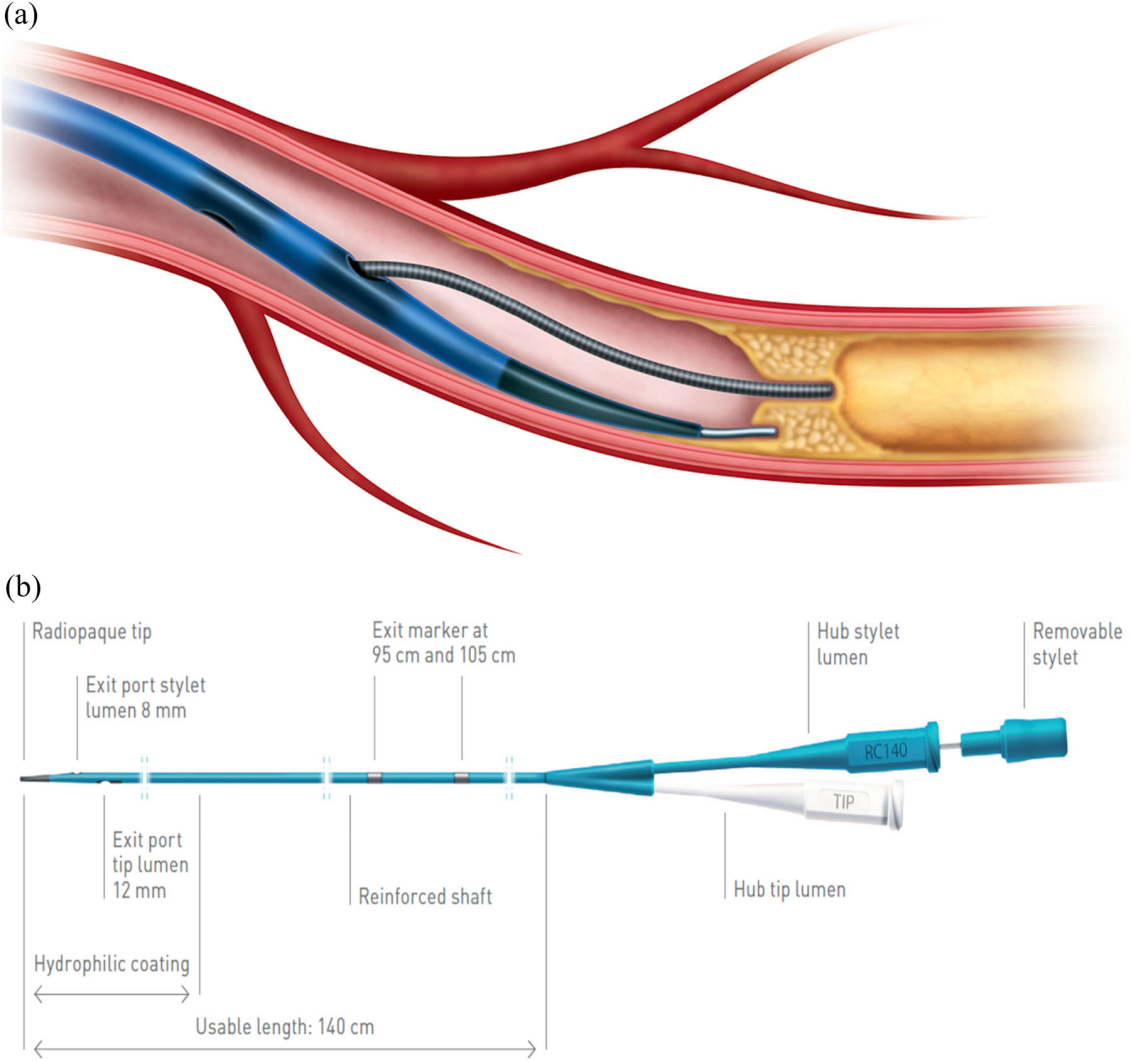
(a) ReCross dual‐lumen microcatheter with wires exiting the distal tip (white) exit port and stylet (blue) exit port. (b) ReCross has a distal tip (lesion entry) profile of 1.5 F. It features a hydrophilic coating, an oval shaft, and proximal shaft profiles of 2.3 and 3.3 F. The device is compatible with 5 F guiding catheters and can be trapped in 6 F catheters. [Color figure can be viewed at wileyonlinelibrary.com]

The ReCross device (IMDS, Roden, The Netherlands) is a uniquely modified true dual‐lumen microcatheter with two over‐the‐wire lumens (Figure 1). Along with the usual distal tip exit port, it features two closely positioned side‐exit ports near its distal end, oriented 180° apart, enabling controlled guidewire redirection [10]. With its unique design incorporating three exit ports, the device holds significant potential for application across all facets of antegrade CTO PCI. Nevertheless, there exists limited published data investigating its efficacy. The objective of our study was to analyze the efficiency and outcome of the ReCross device, following an agreed step‐by‐step protocol in contemporary CTO PCI, based on our firsthand experience.

## 2 Methods

### 2.1 Study Population

During the period between June 2023 and April 2024, we conducted a prospective evaluation of patients undergoing CTO PCI at eight participating centers for potential enrollment. Eligible patients were required to be a minimum of 18 years old, capable and willing to provide informed consent, and scheduled for clinically necessary CTO PCI with a planned primary antegrade approach using an upfront agreed ReCross strategy. Patients were deemed ineligible if the operator intended to use a primary retrograde approach for CTO crossing.

### 2.2 Study Design

The primary objective of the study was to conduct an analysis of the technical success, and patient outcomes associated with the use of ReCross microcatheter during CTO PCI as the upfront primary strategy. Given the limited availability of published data beyond expert opinions, the discretion to employ ReCross as the initial approach was left to the operators.

**Figure 2:**
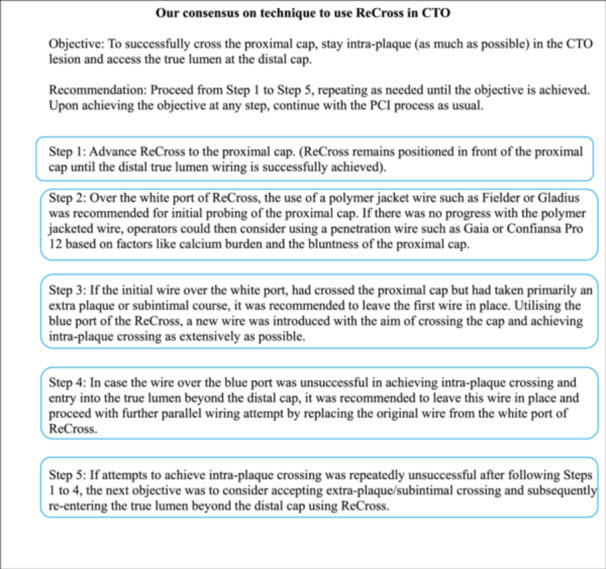
Our consensus on the technique to use ReCross in CTO. [Color figure can be viewed at wileyonlinelibrary.com]

An agreed consensus among the operators on the use of ReCross in tackling CTO for this study is summarized in Figure 2. The TrapIT (IMDS, Roden, The Netherlands) was the preferred device for securing guiding wires within catheters, facilitating an effective exchange of microcatheters.

The selection and sequence of wiring techniques and guidewire choice were completely at the discretion of the operators. In the case of procedural failure, alternative strategies such as antegrade dissection re‐entry (ADR) or retrograde approaches were left to the operators' judgment.

The patients were followed up until their discharge from the hospital, and then either through a clinical visit or a telephone interview. All authors confirm the accuracy and completeness of the data and analyses, as well as the adherence to the trial protocol in this report. Informed consent was obtained from all patients.

### 2.3 Definitions

CTOs were defined as coronary lesions with thrombolysis in myocardial infarction (TIMI) grade 0 flow of at least 3 months duration. Estimation of the duration of occlusion was clinical, based on the first onset of angina, history of myocardial infarction (MI) in the target vessel territory, or comparison with a previous angiogram. Calcification was evaluated through angiography, categorized as mild (spots), moderate (involving 50% of the reference lesion diameter), or severe (involving > 50% of the reference lesion diameter). Vessel tortuosity was defined as moderate when the presence of a minimum of two bends exceeding 70° or a single bend surpassing 90°. In contrast, severe tortuosity is characterized by two bends surpassing 90° or a single bend greater than 120° within the CTO vessel. Blunt stump was defined as the absence of tapering or a funnel shape at the proximal cap, and an ambiguous cap implied that it was not apparent where the proximal cap was located. Interventional collaterals were defined as collaterals deemed feasible for traversal by both a guidewire and a microcatheter at the discretion of the operator. A retrograde procedure was defined as an attempt to cross the lesion through an interventional collateral or a bypass graft that supplies the target vessel distal to the lesion.

Technical success was defined as successful CTO revascularisation with < 30% residual diameter stenosis within the treated segment and restoration of TIMI grade 3 antegrade flow. The occurrence of in‐hospital major adverse cardiac events (MACE) encompassed instances of death, perforations, and tamponade necessitating either pericardiocentesis or surgical intervention before hospital discharge. The Japanese Chronic Total Occlusion (J‐CTO) score calculation adhered to the methodology described by Morino et al. [11].

### 2.4 Study Endpoints

The study's primary endpoint was to evaluate the technical success in contemporary cases. Our hypothesis was that the initial implementation of the ReCross catheter would enhance the success rate of antegrade CTO PCI. The primary safety endpoint focused on procedure‐related mortality, perforations, and tamponades. We postulated that the upfront utilization of ReCross would be linked to a similar incidence of MACE compared to an antegrade wire escalation (AWE) strategy.

The secondary endpoints encompassed (1) the necessity for a change in strategy, (2) the deliverability of the device, (3) the total procedure time, defined as the elapsed time from the administration of local anesthesia for obtaining vascular access to the removal of the last catheter, (4) overall fluoroscopy time, (5) total air kerma radiation exposure, (6) total contrast volume, and (7) the number of wires and microcatheters used.

## 3 Results

A total of 118 patients who underwent CTO PCI across eight centers from June 2023 to April 2024 were included in the study. The majority of cases (94%) were successfully resolved using an upfront ReCross strategy, demonstrating a high level of effectiveness.

### 3.1 Clinical Characteristics

Ninety‐nine (83.9%) of the patients were male, with a mean age of 63 ± 9.95 years. Eight (6.8%) of these patients had undergone a prior coronary artery bypass graft (CABG), and 40 (33.9%) had a history of prior PCI. Twenty‐eight (23.7%) cases represented reattempt at CTO PCI following a previous failed attempt. The clinical characteristics have been summarized in Table 1.

**Table 1:** Baseline clinical characteristics.

	*N* = 118	(%)
Male	99	83.90%
Age (years)	63 ± 9.95	
Previous PCI	40	33.89%
Prior failed attempt	28	23.73%
Previous CABG	8	6.78%
Graft to CTO artery	5	4.24%

Abbreviations: CABG, coronary artery bypass grafting; CTO, chronic total occlusion; PCI, percutaneous coronary intervention.

### 3.2 Angiographic Characteristics

The most frequently targeted vessel was the RCA, accounting for 65.2% of cases, followed by the LAD at 26.3% and the LCx at 8.5%. The majority of lesions were located in the proximal segment (52%), followed by the mid‐segment (42%) and distal segment (6%). Ambiguity in the cap was noted in 9.3% of cases, with 54.2% exhibiting a blunt cap and 36.4% presenting a tapered cap. The majority of lesions had a length exceeding 20 mm (51.69%). All cases exhibited interventional collaterals, with 33.9% having contralateral collaterals, 23.7% demonstrating ipsilateral collaterals, and 42.3% displaying both ipsilateral and contralateral collaterals. Moderate/severe calcification was angiographically observed in 47.5% of cases, while 31.3% exhibited moderate/severe tortuosity. Additionally, a bifurcation at the distal cap was present in 38.9% of cases. The mean J‐CTO score was 2.16 ± 0.95. The angiographic and lesion characteristics are detailed in Table 2.

**Table 2:** Angiographic and lesion characteristics.

	*N* = 118	(%)
Target vessel		
LAD	31	26.30%
LCx	10	8.50%
RCA	77	65.20%
Localization		
Proximal	60	50.90%
Mid	50	42.40%
Distal	7	5.90%
Ostial	1	0.80%
Proximal cap		
Tapered	43	36.44%
Blunt	64	54.24%
Ambiguous	11	9.32%
CTO length (mm)		
1−10	3	2.54%
11−20	54	45.76%
> 21	61	51.69%
Collaterals		
Ipsilateral	28	23.73%
Contralateral	40	33.90%
Ipsi‐ and contralateral	50	42.37%
Calcification (moderate/severe)	56	47.46%
Tortuosity	37	31.36%
In‐stent CTO	5	4.24%
Bifurcation at distal cap	46	38.98%
J‐CTO score		
0	3	2.54%
1	25	21.19%
2	49	41.53%
3	34	28.81%
4	5	4.24%
5	2	1.69%

Abbreviations: CTO, chronic total occlusion; J‐CTO, Japanese chronic total occlusion score; LAD, left anterior descending artery; LCx, left circumflex artery; RCA, right coronary artery.

### 3.3 Procedural and Device Characteristics

The most common guide size utilized was 7 French (81.6%), followed by 8 French (9.2%) and 6 French (9.2%). ReCross achieved a total success rate of 94%, with parallel wiring employed in 47.4%, AWE in 28.8%, and ADR in 17.8% of cases. The success rates for wiring the CTO were comparable between the blue and white ports. In 48 cases (43.6%), ReCross successfully navigated the CTO lesion directly. However, in 12 cases (10.9%), crossing remained unachievable even after pre‐dilatation. The complete procedure and device characteristics are detailed in Table 3 for reference.

**Table 3:** Procedural characteristics.

	*N* = 118	IQR/%
Number of guidewires used	3	(2−4)
Successful guidewire		
Gaia	56	47.45%
Fielder	16	13.55%
Sion	1	0.84%
Gladius	32	27.11%
Confianza	9	7.62%
Others*	4	3.38%
Successful ReCross port		
White port	53	48.18%
Blue port	57	51.81%
ReCross cross the CTO directly	48	43.60%
ReCross cross after pre‐dilation		
No	12	10.90%
Yes	50	45.50%
Recross used for distal bifurcation		
No	93	78.81%
Yes	25	21.19%
IVUS used	59	50.00%

*Note:* Other* ‐ Suoh 03, Hornet, Ultimate Bros, Pilot 200.

Abbreviations: CTO, chronic total occlusion; IQR, interquartile range; IVUS, intravascular ultrasound.

### 3.4 Outcomes and Complications

The detailed procedural outcomes have been summarized in Table 4. Technical success was achieved in 96.6% of the cases. The median procedure time was 90 min, with a fluoroscopic time of 36.5 min and a contrast volume of 160 mL. Intravascular ultrasound (IVUS) was utilized in 50% of the cases, and within these cases, 69.5% revealed evidence of intra‐plaque crossing. The average number of stents used was 2, with a total length of 69.1 mm. There were no procedure‐related mortalities until hospital discharge, although one distal wire perforation complication was reported.

**Table 4:** Outcomes.

	*N* = 118	IQR/%
Successful CTO strategy		
Parallel wiring	56	47.45%
AWE	34	28.81%
ADR	21	17.76%
Retrograde	3	2.54%
Stent deployed?		
No	4	3.38%
Yes	114	96.61%
Number of stents	2	(1−3)
Total stent length (mm)	70.00	(46−96)
Drug‐eluting balloon (DEB)	7	6.14%
Procedure time (min)	90.00	(60−128)
Angiography time (min)	36.50	(27−55)
Radiation dose (mG)	1011.50	(352−1671)
Contrast volume (mL)	160.00	(110−200)
Loss of side branch	1	0.84%
Complications	1	0.84%
Perforation	1	0.84%
Tamponade	0	0.00%
Mortality	0	0.00%

Abbreviations: ADR, antegrade dissection and re‐entry; AWE, antegrade wire escalation; CTO, chronic total occlusion; IQR, interquartile range.

## 4 Discussion

In the absence of randomized trials, the selection of crossing strategies for CTOs primarily depends on various scoring evaluations and the application of the “hybrid algorithm.” Additionally, individual preferences, local device availability, and clinical experience significantly influence decision‐making in this context. AWE stands as the most prevalent CTO strategy, offering distinct advantages in terms of simplicity and widespread accessibility. Notably, AWE serves as the final crossing strategy in approximately 50% of cases across various contemporary CTO PCI registries [1, 3, 11, 12]. Nevertheless, the treatment of more complex CTOs often necessitates the adoption of advanced crossing strategies, such as parallel wiring, dissection or re‐entry techniques, and the retrograde approach.

ReCross microcatheter, by design, presents unique features that set it apart from other dual‐lumen microcatheters. Notably, it features a distal exit port and two lateral ports positioned 180° opposite to each other. This configuration provides the operator with multiple wiring options and allows the simultaneous use of two CTO wires without necessitating any additional maneuvers. It enhances support with anchoring wire, allows the opportunity to repuncture the proximal cap without additional steps, and offers a 360° vessel access capability [10]. In addition to its role in preserving and treating bifurcated coronary branches within or in proximity to the CTO, ReCross enables “modified” parallel wiring, ADR, collateral selection, and managing subintimal hematoma [13]. It is important to note that the ReCross differs from the traditional parallel wiring technique. It utilizes antegrade dual access (ADA) instead of the conventional parallel wiring, which typically involves two single‐lumen microcatheters. This distinction is significant as it eliminates the cumbersome nature associated with the classic parallel wiring approach. This device has a hydrophilic coating and a distal oval shaft, as well as a removable stylet that increases pushability, making it easier to pass through and beyond the CTO body.

Table 5 presents a comparison of recent studies employing mainly antegrade strategy with our own study. The angiographic characteristics and lesion complexity across these studies were found to be comparable. However, direct comparison of the reasons and rates of conversion to alternative strategies such as ADR and retrograde is constrained by inherent limitations, including potential case selection bias and primary intent to treat bias. Nevertheless, these findings offer valuable insights into real‐world experiences with similar cases. In comparison to patients undergoing antegrade‐only procedures, those undergoing retrograde CTO PCI exhibit a higher probability of having a previous history of MI, prior CABG, and peripheral arterial disease [14, 15]. The lesions necessitating the use of retrograde techniques are characterized by greater technical complexity, as indicated by higher J‐CTO scores, longer occlusion lengths, increased calcification, and tortuosity. Consequently, procedures involving retrograde techniques entail prolonged durations, elevated radiation exposure, and increased contrast utilization in comparison to antegrade‐only procedures [16].

**Table 5:** Comparison of trials with pre‐dominantly antegrade approach.

	ReCross	Danek BA, et al.	Karacsonyi J, et al.	Galassi AR, et al.	Nikolapoulos et al.*	L. Azzalini, et al.
	*N* = 118	*N* = 767	*N* = 246	*N* = 1806	*N* = 2023	*N* = 223
Age (years)	63 ± 9.95	65.1 ± 10.3			63.8 ± 9.9	66.3 ± 10.1
Men	83.90%	81.60%			82%	88%
Target vessel						
LAD	31 (26.30%)	29.40%	47 (19.1%)	652 (36.1%)	566 (30%)	45 (20%)
LCx	10 (8.5%)	21.70%	41 (16.6%)	310 (17.1%)	385 (20%)	56 (25%)
RCA	77 (65.20%)	48.80%	158 (64.2%)	786 (43.5%)	903 (47%)	122 (55%)
Proximal cap						
Tapered	43 (36.44%)			910 (50.4%)		
Blunt	64 (54.24%)		108 (43.9%)	876 (48.5%)		106 (48%)
Ambiguous	11 (9.32%)	18.50%	38 (15.4%)		28%	83 (38%)
CTO length (mm)						
1−10	3 (2.54%)					
11−20	54 (45.76%)					
> 21	61 (51.69%)			334 (67.2%)		133 (60%)
Average length	25.05 ± 9.96	28 (16−38)	22 (11−37)	33.6 ± 22.9	24 (15−32)	—
Collaterals		45.6%			46%	55%
Ipsilateral	28 (23.73%)					
Contralateral	40 (33.90%)					
Ipsi‐ and contralateral	50 (42.37%)					
CTO strategy						
AW	90 (76.2%)	519 (67.6%)	124 (50%)	1309 (72%)		
ADR	21 (17.8%)	248 (32.3%)	122 (50%)	497 (27.5%)		
Retrograde	3 (2.5%)			19%		
Calcification	56 (47.46%)	49.10%	108 (43.9%)	466 (25.8%)	42%	103 (46%)
Tortuosity	37 (31.36%)	30.70%	60 (24.39%)	117 (6.4%)	25%	107 (48%)
In‐stent CTO	5 (4.24%)	14.50%	56 (22.7%)	117 (9.8%)		24 (11%)
Bifurcation at distal cap	46 (38.98%)					
J‐CTO score*	2.16 ± 0.95	2.1 ± 1.2	2.05 ± 1.15	2. 1 ± 1.2	2 (1–3)	2.3 ± 1.2
IVUS used	59 (50.00%)	10.70%		287 (15.8%)		
Stent implanted						
No	4 (4%)					
Yes	114 (96.61%)	91.70%				179 (94%)
Number of stents	2 (1−3)	2.3 ± 1.0	2.5 ± 1.1	2.39 ± 1.25		
Total stent length (mm)	70 (46−96)			63.6 ± 33.9		82.1 ± 47.5
DEB	7 (6.14%)					
Technical success	96.61%	93.70%	88.60%	94.70%	88.00%	86%
Procedure time (min)	90 (60−128)	100 (67.5−135.5)	109 (75−185)	102.7 ± 58.8		137 ± 59
Fluoroscopy time (min)	36.50 (27−50)	31.8 (20.1−46.6)	38.5 (24−66)	41.2 ± 29.3		49.6 ± 21.8
Radiation dose (mG)	1011.5 (352−1671)	2600 (1600−4200)	2260 (1230−3910)			
Contrast volume (mL)	160 (110−200)	245 (180−318.3)	255 (155−350)	310.9 ± 238.8		373 ± 143
Side branch loss	1 (0.84%)		15 (6.0%)			
Any complications	1 (0.84%)	MACE 1.1%	MACE 3.65%		MACE 1.1%	6 (2.7%)
Perforations (any)	1 (0.84%)	0.03%	15 (6.0%)	49 (2.7%)		
Mortality	0 (0.00%)	0.1%	3 (1.21%)	2 (0.1%)	10 (0.4%)	4.7%

Abbreviations: ADR, antegrade dissection re‐entry; AW, antegrade wiring (includes all antegrade wiring techniques such as antegrade wire escalation and parallel wiring); CTO, chronic total occlusion; DEB, drug eluting balloon; IVUS, intravascular ultrasound; LAD, left anterior descending artery; LCx, left circumflex artery; RCA, right coronary artery.

*Nikolapoulos et al. [11].

In recent years, it is evident that modern CTO PCI procedures have demonstrated improved safety and efficiency while maintaining technical success rates. Additionally, data from the PROGRESS‐CTO registry shows a significant drop in the retrograde approach from 39% (2012−2016) to 29% (2017−2019) [14, 15, 16]. Despite a reduction in the use of retrograde techniques, there has been no discernible impact on the rates of technical and procedural success. Moreover, there has been a marked decrease in in‐hospital MACE, procedure duration, as well as the use of radiation and contrast [16].

The success rate using the ReCross device in an open‐label registry from the UK involving consecutive patients undergoing CTO PCI with ReCross was found to be close to 95%, even when necessitating conversion to ADR [17]. Their study indicated that ReCross was utilized for re‐entry in 29% of cases, with a success rate of 73% in those instances. The analysis of cases where controlled re‐entry failed revealed that the primary causes were the inability to puncture the true lumen and, in approximately 10% of cases, failure to deliver the ReCross. The findings of our study align with these results, demonstrating that ReCross enhances the likelihood of success with the antegrade technique and is applicable for controlled re‐entry in ADR cases.

The average procedure time observed in our study was notably lower in comparison to other similar studies (Table 5). We attribute this increased efficiency and high success rate in our study to the algorithmic utilization (Figure 2) of the ReCross device. Furthermore, we conducted a detailed analysis of our cases to identify factors influencing procedure time. We categorized all cases into two groups, namely “< 60 min” and “> 60 min,” to ascertain differences between them. The findings (Table 6a) revealed a correlation between an increased J‐CTO score and longer procedure times, with a higher utilization of IVUS observed in the “> 60 min” group. Notably, an in‐depth review of cases exceeding 90 min, as depicted in our analysis (Table 6b), unveiled that a higher calcium burden, longer lesions, and higher J‐CTO score were associated with prolonged procedure durations. Additionally, there was an increased utilization of guidewires and a greater number of stents associated with prolonged procedure durations.

**Table 6a:** Comparison of cases completed in “< 60 min” and “> 60 min.”

	< 60 min (*N* = 26)	> 60 min (*N* = 92)	*p* value
J CTO score	1.54 (± 0.76)	2.34 (± 0.92)	< 0.01
CTO length	22.15 (± 6.64)	25.86 (± 10.59)	0.093
IVUS used	5 (19.2%)	54 (58.7%)	< 0.01

*Note:* Both groups were compared using either the Fisher exact test or the chi‐square test. A *p*‐value of less than 0.05 was regarded as statistically significant.

**Table 6b:** Comparison of cases completed in “< 90 min” and “> 90 min.”

	< 90 min (*N* = 58)	> 90 min (*N* = 60)	*p* value
CTO length	22.03 (±7.68)	28.1 (±11.0)	0.001
J‐CTO score	1.72 (± 0.84)	2.62 (± 0.83)	< 0.001
Number of guidewires	2.23 (± 0.99)	3.56 (± 1.28)	< 0.001
Number of stents	1.75 (± 0.78)	2.4 (± 0.77)	< 0.001
Total stent length	56.01 (± 28.27)	82.72 (± 27.11)	< 0.001

*Note:* Both groups were compared using either the Fisher exact test or the chi‐square test. A *p*‐value of less than 0.05 was regarded as statistically significant.

In terms of deliverability, only 12 (10.9%) cases in our study experienced difficulty in delivering the ReCross device, even after pre‐dilatation. Moreover, ReCross requires a minimal learning curve to optimize its use, potentially leading to cost reductions when implemented as an upfront strategy. The new algorithm for the use of the ReCross microcatheter in this study has proven effective and successful in our initial experience, providing a foundational basis for broader clinical trials.

### 4.1 Study Limitations

Our study constituted a prospective analysis of patients who underwent CTO PCI utilizing ReCross in high‐volume centers, overseen by proficient operators. While the implementation of ReCross as an initial approach shows promise, it is imperative to consider that the success observed may be attributable to the considerable expertise and discerning case selection and strategies adopted by highly experienced operators. While acknowledging the potential for interobserver variability, it should be noted that all operators involved in this study were highly experienced and performed a high volume of CTO procedures. The ReCross device, while versatile, is not the definitive solution for antegrade CTO PCI. While it can enhance the likelihood of success, it has inherent limitations, like any other device. Operators must possess comprehensive skills in all aspects of CTO PCI to optimize the likelihood of success.

## 5 Conclusions

Our initial experience with the ReCross microcatheter suggests that, in selected cases and with the application of a dedicated algorithm, the anterograde approach can be highly successful. This success was achieved through a broad repertoire of techniques, including AWE, conventional or parallel wiring techniques, and ADR. However, further studies are needed to validate these findings on a larger scale.

## Clinical Perspectives

Safety and efficiency are key in CTO PCI. While ReCross offers unique benefits in antegrade CTO PCI, data on its overall effectiveness is limited. Our algorithm using ReCross has shown improved success and efficiency, but more research is needed to confirm these results on a larger scale.

## Conflicts of Interest

Mohamed Ayoub reports consulting/speaker/proctoring institutional honoraria from Asahi Intecc, Biotronik, Boston Scientific, Cardinal Health, Cordis, Medtronic, Teleflex, Terumo, IMDS and Abiomed. Abdul Mozid has received speakers fees from IMDS and Biotronik. Xavier Millan has received honoraria and consulting fees from Biotronik. Claudiu Ungureanu reports consulting/speaker honoraria from Abbott Vascular, Biotronik, Boston Scientific, IMDS, Medtronic, Top Medical, and Teleflex; research support: Boston Scientific, Abbott Vascular. The institution of Johan Bennett receives a research grant from Shockwave IVLS, Johan Bennett receives institutional consulting fees from Biotronik AG, Elixir and Boston Scientific, speaker fees/honoraria from Biotronik AG, Elixir, Boston Scientific and Abbott Vascular, participates in the advisory boards of Boston Scientific and Elixir, and has a leadership or fiduciary role for Biotronik. Alexandre Avran reports proctoring for Boston Scientific, Asahi, Abbott, and Biotronik. The other authors declare no conflicts of interest.

## Data Availability

Data will be made available from the authors on reasonable request.
